# Ireland and Medical Benefit

**Published:** 1913-03-15

**Authors:** 


### IRELAND AND MEDICAL BENEFIT.

When the National Insurance Act was introduced and passed, a special provision was included by which insured persons in Ireland were to receive no medical benefit, and were to pay on a reduced scale of contributions because of the omission. The cause of this differential treatment of Ireland was the existence of what is known in that country as the dispensary system, by which local authorities (under a rather indefinite amount of control from the Irish Local Government Board) provide and pay doctors in each district whose duty it is to render medical service to the least affluent of the population. This system is essentially somewhat similar to that of the Poor-Law medical service in Great Britain. But whereas in Ireland the bulk of the rural wage-earners, and a good many of the urban, were thus clients of a rate-paid doctor, in this country only a minute fraction were thus provided for.

It was accordingly thought best not to meddle with the existing system in Ireland, especially as the Insurance Act does not make any provision for medical benefit for the dependants of insured persons. But it now appears that many—probably a majority—of the insured in Ireland are in favour of paying the extra contributions and receiving " medical benefit " in return, just as their fellow-workers in Great Britain already do. A Treasury Committee is touring Ireland and taking evidence in the chief centres concerning the desirability of adopting this plan. It appears to be agreed that the urban districts favour it strongly, but that the peasantry are not so keen about it, because in the numerous sparsely populated districts where there is only one doctor in reach it means paying for his services instead of getting them for nothing. In the nature of things, the intelligent, well-paid, well-organised townsmen can get their views presented more ably than the scattered, disorganised, and more indigent rural labourers. But, even allowing for that, it is certain that the bulk of the Irish insured wish for " medical benefit "; and probably the Treasury Committee will recommend that it shall be granted.

The medical profession in Ireland, as represented by a spokesman on behalf of the Irish and the British Medical Associations, is neutral in the matter; and is willing to co-operate whole-heartedly on certain terms. The latter include administration of medical benefit through Insurance Committees, not through Friendly Societies, free choice of patient and doctor, and an income limit of £2 per week. We can well understand that the profession in Ireland, especially in rural areas, is in the mood for giving medical benefit a trial; for the lot of the dispensary doctor is so uncommonly hard at present that any change would be almost bound to be an improvement. The dispensary doctors are, in the main, grossly underpaid for their work; and the inevitable result is that many of them get into the habit of rendering slack and perfunctory attention to their patients. Could the Insurance Act bring better pay, and exact better work in consequence, it would be for the good of the profession no less than that of the insured classes. But the number of " ifs " is ominous.

